# Neuroendocrine Lung Cancer Mouse Models: An Overview

**DOI:** 10.3390/cancers13010014

**Published:** 2020-12-22

**Authors:** Corina Lorz, Marta Oteo, Mirentxu Santos

**Affiliations:** 1 Molecular Oncology Unit (CIEMAT), Institute of Biomedical Research, University Hospital “12 de Octubre”, CIBERONC, 28040 Madrid, Spain; clorz@ciemat.es; 2 Biomedical Applications and Pharmacokinetics Unit (CIEMAT), 28040 Madrid, Spain; marta.oteo@ciemat.es

**Keywords:** mouse models, lung cancer, typical carcinoid, atypical carcinoid, large cell neuroendocrine lung cancer, small cell lung carcinoma, genetic landscape, molecular imaging

## Abstract

**Simple Summary:**

Neuroendocrine lung tumors are a heterogeneous group of malignancies that share a common neuroendocrine nature. They range from low- and intermediate-grade typical and atypical carcinoma, to the highly malignant large cell neuroendocrine lung carcinoma and small cell carcinoma, with marked differences in incidences and prognosis. This review delineates the current knowledge of the genetic landscape of the human tumors, its influence in the development of genetically engineered mouse models (GEMMs) and the molecular imaging tools available to detect and monitor these diseases. While small cell lung carcinoma is one of the diseases best represented by GEMMs, there is a worrying lack of animal models for the other members of the group, these being understudied diseases. Regardless of the incidence and material available, they all are in urgent need of effective therapies.

**Abstract:**

Neuroendocrine lung tumors comprise a range of malignancies that extend from benign tumorlets to the most prevalent and aggressive Small Cell Lung Carcinoma (SCLC). They also include low-grade Typical Carcinoids (TC), intermediate-grade Atypical Carcinoids (AC) and high-grade Large Cell Neuroendocrine Carcinoma (LCNEC). Optimal treatment options have not been adequately established: surgical resection when possible is the choice for AC and TC, and for SCLC chemotherapy and very recently, immune checkpoint inhibitors. Some mouse models have been generated based on the molecular alterations identified in genomic analyses of human tumors. With the exception of SCLC, there is a limited availability of (preclinical) models making their development an unmet need for the understanding of the molecular mechanisms underlying these diseases. For SCLC, these models are crucial for translational research and novel drug testing, given the paucity of human material from surgery. The lack of early detection systems for lung cancer point them out as suitable frameworks for the identification of biomarkers at the initial stages of tumor development and for testing molecular imaging methods based on somatostatin receptors. Here, we review the relevant models reported to date, their impact on the understanding of the biology of the tumor subtypes and their relationships, as well as the effect of the analyses of the genetic landscape of the human tumors and molecular imaging tools in their development.

## 1. Introduction

Neuroendocrine lung tumors account for approximately 20% of all lung cancers [1]. They arise in the lung unlike other neuroendocrine tumors with other anatomical body localization (mainly small intestine and rectum) [2]. Since the 2015 World Health Organization (WHO) classification they are grouped together in one category, including typical carcinoid (TC), 1.8% of lung cancers; atypical carcinoid (AC), 0.2% of the cases; large cell neuroendocrine carcinomas (LCNEC), 3%; and small cell lung carcinoma (SCLC), accounting for 15% of lung cancer cases [1,3,4]. They comprise a range of malignancies with markedly different traits, incidences, aggressiveness, prognosis, and management. All of them share histologic characteristics (an organoid architecture and growth pattern, with rosettes and peripheral palisading), while they differ in their proliferation count, necrosis, cell size, and nuclear features. While LCNEC and SCLC are high-grade aggressive tumors, TC and AC are well-differentiated tumors of low- and intermediate-grade malignancy, respectively, that nonetheless metastasize with a rather high frequency (10–23% of TC and 40–50% of AC) [1]. Lung low- and intermediate-grade neuroendocrine malignancies TC and AC are also known as carcinoids, which differentiate them from the WHO classification proposed for neuroendocrine tumors arising in other anatomical locations [3].

**Figure 1:**
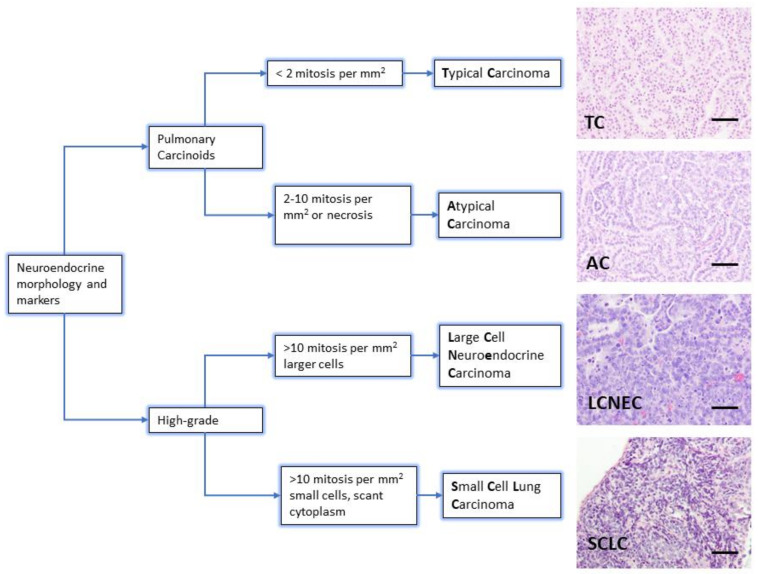
Lung neuroendocrine tumor types. WHO morphologic criteria for classification of lung neuroendocrine tumors. Pulmonary Carcinoids (Typical Carcinoma, TC and Atypical Carcinoma, AC) are separated from high-grade tumors (Large Cell Neuroendocrine Carcinoma, LCNEC and Small Cell Carcinoma, SCLC) according to proliferation count (mitosis) [3]. Right column shows a representative hematoxylin and eosin staining from lung tumors arisen in Genetically Engineered Mouse Models of each quoted tumor type [21,22]. Scale bars, 50 μm.

The diagnosis of neuroendocrine lung tumors relies upon histological examination and immunohistochemical detection of general markers of neuroendocrine differentiation such as chromogranin A, synaptophysin, neuroendocrine cell adhesion molecule (NCAM) and insulinoma associated protein 1 (INSM1). These markers provide a reliable profile to confirm their neuroendocrine nature [3,5,6,7]. However, they do not distinguish among the different subtypes of neuroendocrine tumors. Histologically, the presence of mitosis separates the groups: 2/mm^2^ for TC and 2–10/mm^2^ for AC, >10/mm^2^ for LCNEC and SCLC. Presence of necrosis is a feature of high-grade tumors, and LCNEC and SCLC are separated on the basis of cytological criteria (cell size and nuclear features) [3] (Figure 1). Ki-67 (a marker of proliferating cells spanning from G1 to M phase) labelling index is emerging as a discriminator between carcinoids and SCLC that helps for clinical decision making, particularly on cytology samples for non-resectable cases [5]. 

With regard to prognosis, patients with TC have a better prognosis than those with AC, and both have a better outcome than patients with LCNEC or SCLC that show lower survival rates (40% and 35% 5-year survival rate, respectively) [8]. Surgical resection is the treatment of choice in patients with localized TC or AC [9] and the only curative option for lung neuroendocrine tumors. When carcinoids have metastasized, they are difficult to treat due to high resistance to radiotherapy and chemotherapy. Peptide receptor radionuclide therapy (PRRT) alongside everolimus has been recently proposed for patients with advanced TC or AC [10]. For patients with LCNEC and SCLC, resection is only employed in the very few cases detected before metastatic spread at the time of diagnosis. For SCLC, chemotherapy in conjunction with radiotherapy is the standard of care, but this is only transiently effective. Immunotherapy is emerging as an alternative but only for a limited number of cases [11]. Responsiveness to SCLC chemotherapy regimens has been reported in some LCNEC series [12] but this is not a consistent finding. The optimal treatment for LCNEC has not been adequately established. There is a lack of consensus for treatment guidelines, which further complicates their management [9,13]. Overall, neuroendocrine lung tumors are in need of additional and novel effective (systemic and targeted) therapies.

As mentioned above, incidences vary dramatically among the different groups, and have changed over the past few years. A decrease has been reported for SCLC, while the annual incidence for carcinoids and LCNEC has increased worldwide, particularly at the advanced stages [2,9,14,15], yet they remain rare. With the exception of SCLC, which accounts for ≈15% of all lung cancers, neuroendocrine lung tumors present a limited incidence. This low incidence is one of the main reasons why a comprehensive analysis of their molecular and genomic alterations remains elusive, although recent efforts have been made in performing genomic studies in the rare neuroendocrine lung tumors (and SCLC) [1,16,17,18,19,20]. 

To circumvent this limitation, mouse models have emerged as powerful and necessary tools to delineate the development, progression, and behavior of human tumors, and to facilitate clinical application of novel therapies in patients. Another significant challenge in neuroendocrine lung cancer research is the scarcity of patient samples due to the limited incidence of the disease or owing to the fact that most diagnoses and clinical decisions are based on fine-needle aspirates or small biopsies. Therefore, mouse models, including Genetically Engineered Mouse Models (GEMMs), patient-derived and human cancer cell line xenografts, are invaluable tools to advance our understanding of the underpinnings of these diseases and to carry out translational research, identifying vulnerabilities that can potentially be targeted. 

## 2. Genetic Landscape of Human Neuroendocrine Tumors

Human high-grade neuroendocrine tumors display a nearly universal loss of function (gene mutation, copy number loss or methylation) in either or both the tumor suppressors TP53 (which encodes for p53) and RB1 (which encodes for retinoblastoma, RB1) [16,17,18,23,24,25]. TP53 inactivation is a common event in neuroendocrine and non-neuroendocrine lung tumors; however, RB1 loss of function is very characteristic of the former. SCLC is the best molecularly characterized neuroendocrine tumor. The first attempts to identify SCLC subclasses based on their mutational profile [16,17,18] were not able to yield clear SCLC subtypes due to the abovementioned uniform genomic inactivation of TP53 and RB1, and to failure of other recurrent mutations (such as EP300, CREBBP, TP73, COL22A1, NOTCH1, MLL) to show reliable co-occurrence or mutual exclusivity. Interestingly, epigenetic and gene expression studies unveiled the existence of different subtypes of SCLC based on their relative expression of the neuroendocrine lineage transcription factors ASCL1 (achaete-scute homologue 1) and NeuroD1 (neurogenic differentiation factor D1) and other transcription factors [26]. The existence of four subtypes has been confirmed by different studies [27,28,29] and a nomenclature for SCLC molecular subtypes has recently been proposed [30]. SCLC-A is the major group and displays high expression of ASCL1 (and its transcriptional target DLL3) and low expression of NeuroD1 markers. SCLC-N is the second most frequent subtype, and is characterized by high NeuroD1 and low (albeit variable) ASCL1 gene expression. SCLC-Y and SCLC-P represent smaller groups and comprise ASCL1/NeuroD1/INSM1 (insulinoma-associated protein 1, a neuroendocrine differentiation-promoting transcription factor) low tumors. These two subgroups, referred to as non-neuroendocrine, double negative or SQ-P (resembling primitive squamous tumors) by different studies [26,27,30] are characterized by the expression of the transcriptional co-activator YAP1 and the transcriptional factor POU2F3, respectively [28,29,30]. Thus, it has become apparent that although loss of function of TP53 and/or RB1 is required for SCLC development, the existence of different subtypes is governed by gene expression programs under the control of specific transcriptional regulators.

Genomic profiling of LCNEC tumors has shown frequent mutations in TP53 and RB1, although the prevalence of RB1 mutations is lower than in SCLC [24]. Recently, the first comprehensive characterization of LCNEC, combining sets of gene mutations with patterns of gene expression and comparing them to those in SCLC [23], highlighted differences and commonalities between the two types of high-grade neuroendocrine tumors. This study identified two molecular subgroups within LCNEC, termed type I and type II. Type I LCNEC displayed frequent mutations in TP53 and two other genes, STK11 (also known as LKB1, which encodes for a serine/threonine kinase that regulates cell polarity) and KEAP1 (involved in oxidative stress response), but not in RB1. Type II LCNEC bore frequent mutations in TP53 and RB1, and from that standpoint one would expect it to be the most similar to SCLC. In fact, two major LCNEC subsets had previously been identified [25] based on their profile of altered genes, one considered SCLC-like, with frequent TP53/RB1 mutations, and the other nonSCLC-like, with frequent TP53/STK11/KEAP1/KRAS mutations. However, transcriptional profiles of type I and II LCNEC indicated the opposite [23]. Type I LCNEC showed high levels of expression of ASCL1 and DLL3, similar to SCLC-A (ASCL-high/NeuroD1-high) tumors, and low levels of NOTCH. Conversely, type II LCNEC had reduced expression of ASCL1, DLL3, and other neuroendocrine genes, and high expression of NOTCH and immune-related pathways.

In contrast to high-grade neuroendocrine carcinomas, well-differentiated neuroendocrine tumors TC and AC show low mutation rate and rare mutations in TP53 and RB1 genes. Pulmonary carcinoids display frequent alterations in chromatin-remodeling genes such as MEN1 (the most frequently mutated), ARID1A and components of the SWI/SNF complex [19,20,31]. Comprehensive multi-omic analysis identified different molecular subgroups within well-differentiated neuroendocrine tumors [20]. Molecular group A (further divided into A1 and A2) consisted mainly of TC; group B, enriched in AC; and supra-carcinoids, with very few samples. While high-grade neuroendocrine tumors and well-differentiated carcinoids possibly represent different diseases and not just a range of tumors with common pathogeneses, these comprehensive studies reveal the existence of molecular links between both groups of lung neuroendocrine tumors. Thus, the A1 subgroup of pulmonary carcinoids showed high expression of ASCL1 and DLL3 genes, which is similar to SCLC-A and type I LCNEC. The supra-carcinoid subgroup displayed molecular characteristics similar to LCNEC, including high expression of immune checkpoint genes. In fact, one case of LCNEC clustered in this group [20]. This observation has led to the suggestion that supra-carcinoids could represent the lung equivalent to grade-3 neuroendocrine tumors present in other organs; currently this grade is only considered for the high-grade lung neuroendocrine SCLC and LCNEC [20,32].

## 3. Modelling Neuroendocrine Lung Cancer with GEMMs

Mouse models of neuroendocrine lung cancer based on patient-derived mutations are used to determine the mechanisms by which the disease arises and progresses. Advances in genetics, sequencing, and transcriptomic studies have identified disease-associated genetic alterations in neuroendocrine tumors [17,19,20,23,25]. To determine causality between genetics and disease, accurate models for molecular dissection are required.

GEMMs are essential tools to delineate the multistage pathogenesis of human tumors, behavior, development and extension, progression, and therapy response. GEMMs can be used to induce spontaneous tumor growth via conventional transgenic or conditional mechanisms in a native immunocompetent microenvironment. Transgenic mice are generated by microinjection of DNA into the pronucleus of zygotes or injection of embryonic stem cells into blastocysts, to produce the desired loss, gain of function mutations, or substitutions (knock out or knock in). The currently available tools allow us to constitutively or conditionally activate or inactivate genes in distinct cell types at any desired moment. So far, the most widely used strategy in modeling neuroendocrine lung cancer is based on the ability of cre recombinase to direct site-specific DNA recombination between pairs of *loxP* sites. The use of fluorescent reporters that mark the switched cells helps to track and isolate cancer cells as tumor growth progresses from initial lesions to metastasis. Bioluminescent reporters also enable us to conduct lineage tracing, monitoring and quantification of the tumor in situ [33,34,35]. A drawback of current mouse models that harbor multiple genetic modifications is the time investment required to breed mice to generate such complex genotypes. This costly process involves hundreds of animals and takes years. Recent advances, including CRISPR/Cas9, have been used in vivo to manipulate specific genes and specific populations of cells [36,37] thus allowing faster generation of GEMMs which develop SCLC. Hopefully, additional neuroendocrine lung cancer models will soon be available by means of these techniques.

Another point to consider is that human tumors usually harbor a high burden of point mutations caused by cigarette smoke exposure, while tumors in GEMMs develop mostly due to targeted gene rearrangements. At any rate, such models serve to distinguish passenger from driver mutations. Exposure to chemical carcinogens (including smoke exposure) was one of the main focuses of the early lung cancer studies using mouse models, that lead to the development of adenocarcinoma in the majority of cases [38,39]. However, neuroendocrine lung tumors are virtually never found in spontaneous or chemically induced lung cancer models. This could be explained at least in part because most of the carcinogens, for which the mechanism of action is known, induce the activation of oncogenes such as K-ras [40,41,42], while the combinations of *Trp53* and *Rb* alterations, responsible for the development of high-grade neuroendocrine tumors, are almost never found. Nevertheless, when applied to Rb-family deficient GEMMs, chemical carcinogens induce exclusively neuroendocrine tumors [21]. 

Some lung tumors modelled in mice accurately mimic their human counterparts [33], and one possible explanation resides in the feasibility of a refined method to neatly access mouse lungs: intratracheal infection of adenovirus in adult lungs has proven to be a robust method for modelling lung cancer [43]. Another contributing factor is the availability of specific cell promoters that determine the cell-type specificity of the genetic modification. Specific cell promoters are active in the different lung cell types: the rat CGRP promoter was identified as a neuroendocrine cell-specific promoter [44]; the 3.7-kb fragment of the human SPC promoter activity is restricted to alveolar type 2 (AT2) cells [45]. Similarly, Aquaporin 5 (Aqp5) promoter activity is constrained to alveolar type I cells [46]; the mouse Scgb1a1 (Secretoglobin1a1, also known as CCSP, CC10, and CCA) promoter fragment mainly targets bronchiolar Clara cells [47]. Although not expressed exclusively in the respiratory system, Keratin 5 (K5) and Keratin 14 (K14) promoters have been used to target basal cells in the airway epithelium [22,48]. Similarly, a 1-kb fragment of the human FOXJ1 promoter directs reporter gene expression to all ciliated cells including those of the lung, oviducts, ependyma, and testis [49].

### Cell of Origin of SCLC

The utilization of adenoviruses carrying different promoters to direct cre expression to specific epithelial cell types has been particularly successful in dissecting the cell of origin of SCLC. This type of data can only be inferred from animal models. As different promoters targeting specific populations in the lung epithelia are employed in the construction of distinct adeno-cre viruses, genetic recombination is achieved in a cell-type restricted manner. Thus, SCLC has been reported to arise mainly from neuroendocrine cells (using a CGRP promoter) [50,51], and recent work using a neuroendocrine specific driver (Ascl1^creERT2^) showed that SCLC tumors can arise from a small subpopulation of rare, differentiated neuroendocrine stem cells [52]. However, there is strong evidence supporting that SCLC tumors can arise from other cell types in the lung. It is of note that the use of viruses carrying the SPC promoter, which targets AT2 cells, and K5 and K14 promoters that target keratin K5 and keratin K14 expressing epithelial basal cells, respectively, render SCLC [22,50,53]. In contrast, Sutherland et al. [50] reported no tumor formation when using the CC10 promoter that targets Clara cells. Taken together, data gathered from animal models support the idea that non-neuroendocrine lung epithelial cells may also serve as the cell type of origin for SCLC, at least in specific genetic contexts [22,50,53]. This suggests high cellular plasticity and an interplay—at least in mice—between targeted cells and modified genes. The question of the extent to which this is the case in humans remains open. Otherwise, in spite of the available tools, the cell(s) of origin of the other neuroendocrine lung tumor types (TC, AC and LCNEC) remain unknown. 

Specifically targeting genetic alterations to different cell types has not only served the purpose of identifying the cell of origin from which SCLC arises, but also has revealed the existence of different metastatic programs [54], an activating or suppressive role of FGFR1 [53], and determine that the same set of genetic alterations can generate different types of high-grade neuroendocrine tumor [22,55]. A thorough review of the cells of origin of lung cancer and its influence in inter- and intratumoral heterogeneity has recently been published [56]. 

## 4. Animal Models of Pulmonary Carcinoids

There is a notable paucity of biological material for the study of these rare tumors, which has impeded significant advances in patient treatment. The studies of tumor biology using pulmonary carcinoids have been hindered by a lack of in vitro and in vivo models representing the carcinoid phenotype and behavior. Hardly a handful of different typical carcinoid cell lines have been generated [57], and two of them (NCI-H720 and NCI-H727) give rise to tumors when orthotopically injected into mice [58]. NCI-H727 [59] is the most used cell line for the study of typical carcinoma. This cell line has been used to test several compounds or molecular imaging techniques in different studies [60,61,62,63,64,65]. However, the characteristics of the primary tumors originating from this cell line are not consistent with the biology of a typical carcinoid, thus hampering the understanding of the molecular mechanisms underlying this tumor type. Chemically induced GEMMs for TC and AC exist: a triple knockout mutant model in which all the three Rb family members are ablated develops TC after DHPN (N-bis(2-hydroxypropyl) nitrosamine, a potent mutagen and a wide-spectrum carcinogen in rodents) administration and AC after urethane (an inducer of K-ras activation) treatment [21].

Thus, as mentioned above, and although the worldwide incidence of pulmonary carcinoids is increasing, modeling these tumor types in the mouse is still in its infancy [60] and implies an unmet need for studying the biology and novel intervention strategies for these tumors. Given the mutational pattern of MEN1, ARID1, EIF1AX, the SW1/SNF complex, ATM, PSIP1, and ROBO1 reported in human low-grade neuroendocrine tumors [19,20], it would be worthwhile to generate lung preclinical models based on these genetic changes aiming to recapitulate the human types and serve for the development and testing of new therapeutic approaches, particularly for the pulmonary carcinoids that metastasize and subsequently worsen their prognosis. In fact, several MEN1 conditional and knockout models have been generated that mimic neuroendocrine disorders and tumors in other organs different from the lung such as pancreas, thyroid, adrenal, and pituitary glands [60]. Combining approaches that target specific cell types in the lung with the introduction of the appropriate genetic changes found in human pulmonary carcinoids, the scientific community should be able to generate TC and AC mouse models resembling their human counterparts. These models will undoubtedly allow progress in the understanding of these understudied diseases.

## 5. Animal Models of LCNEC

A defined GEMM model of LCNEC based on the ablation of four tumor suppressors (*Rb, Rbl1, Pten, Trp53*) in a wide variety of lung epithelial cell types (using the CMV promoter) has recently been described [22,55]. It is interesting to note that, in this quadruple mutant mouse, differential development of LCNEC or SCLC resides in the targeted cell initiating tumor progression. In this model, restricted cell type targeting to basal cells gives rise predominantly to SCLC, but a spectrum of neuroendocrine tumors is observed [22] suggesting a close relationship between both tumoral types. Along these lines, in 2015, Gazdar et al. [66] published a thorough histopathological examination of relevant mouse models of SCLC. They found that most of the models evaluated depict the whole spectrum of high-grade neuroendocrine lung tumors, and in some stages of development the LCNEC component was predominant over the SCLC component. They also reported both components to be often intimately intermixed, suggesting high plasticity and transition from one to the other [38,66]. Akeno et al. also reported the formation of LCNEC along with SCLC in a *Trp53* mutant and deficient *Rb1* mouse [67]. However, the quadruple *Rb, Rbl1, Pten, Trp53* mutant mice represent the first and only mouse model for LCNEC reported so far [22,56].

## 6. Animal Models of SCLC

In sharp contrast with the other types of pulmonary neuroendocrine tumors, a number of GEMMs for SCLC have been successfully reported. These tumors faithfully reproduce the histopathological features of the human condition (and the neuroendocrine nature of the tumor) [34].

Based on the fact that p53 and RB1 are almost always inactivated in patients with SCLC, Meuwissen et al. [68] generated the first mouse model in which conditional deletion of *Rb1* and *Trp53* was accomplished by intratracheal adenoviral delivery of cre using the ubiquitous CMV promoter. The sporadic inactivation of both genes in a wide variety of lung epithelial cells renders (after long latency periods) spontaneous tumors that closely mimic the histopathology and metastatic behavior of human patients. This basic model was subsequently followed by variant versions when additional genetic modifications found or suspected in human SCLC tumors were introduced: inactivation of *Rbl2*, another member or the Rb family, resulted in accelerated SCLC tumor development [69]. This triple *knock out Rb1/Trp53/Rbl2* maintains the histologic features and metastatic pattern of the disease. Loss of *Pten*, a gene frequently mutated in human SCLC, accelerated tumor progression as described by two groups: McFadden et al. [51] reported SCLC tumor development when using the neuroendocrine-specific CGRP promoter; meanwhile, Cui et al. [70] observed a mixture of SCLC/LCNEC/ADC when using the CMV promoter. Combined deletion of two members of the retinoblastoma family, *Rb1* and *Rbl1,* together with *Pten* and *Trp53,* resulted in a dramatic acceleration of SCLC tumor progression when epithelial basal cells were targeted by using the keratin K5 promoter [22].

Overexpression of Mycl, a transcription factor member of a family of oncogenes which was found amplified in ~9% of human SCLC [17] together with the targeted deletion of *Rb1* and *Trp53* showed an earlier onset of SCLC and shortened latency of tumor development. In this model, metastasis is rarely seen [71,72]. However, a mutant Myc^T58A^ dramatically accelerates tumorigenesis and metastasis in the *Rb1/Trp53* null SCLC promoting a variant phenotype [73]. These “myc models” also evidence the SCLC tumor heterogeneity. Similarly, overexpression of Nfib together with biallelic inactivation of *Rb1* and *Trp53* promoted earlier onset of SCLC and enhanced metastasis [74,75]. These triple or quadruple mutant models reduce the long latency periods of the first reported *Rb1/Trp53* deficient mice, as they introduce the additional secondary genetic changes necessary for the tumor development. The role of Notch as a tumor suppressor in SCLC was established, as in the Rb1/Trp53/Rbl2 background there was a significant reduction of the number of tumors that developed when an activated form of NOTCH1 and NOTCH2 was overexpressed [17]. Later on, subsequent models showed that Notch exerts its suppressor activities through different mechanisms [36,52]. 

These models have been invaluable tools to understand the biology of SCLC, for its preclinical intervention and translational research. Contrary to the other neuroendocrine lung tumor types, they make of SCLC one of the best represented diseases by GEMMs for the study of human cancer [30,38] (Table 1). Gazdar et al. [38,66] also remark on the importance of these GEMMs in providing the opportunity to study the early stages and the multistage pathogenesis of the disease, as SCLCs are seldom resected, thus hindering the opportunity of observing initial or preinvasive lesions in humans. These GEMMSs also reproduce the hepatic, mediastinal, and lymph node invasion seen in humans.

### A Recent Classification of Subtypes of SCLC 

Until recently, SCLC were considered a relatively homogenous lung cancer type. To date the WHO classification recognizes only one form of SCLC (in current clinical practice). This has led to similar chemotherapy treatment for patients in the last decades [78]. However, and as stated above, recent studies have reported a considerable amount of heterogeneity among SCLCs, mostly based on the studies that identify subtypes based on the expression of lineage-specific transcription factors. They also take into consideration expression of neuroendocrine markers [27], growth properties in cultures [79] and amplification or expression of MYC [73]. Animal models have a role in defining these subtypes by complementing data from cell lines and human tumors. The role of Ascl1 (and NeuroD1) in developing tumors of the Ascl1^high^ (NeuroD1^low^) subtype was first reported in a mouse model generated by adding the inactivation of *Ascl1* or *Neuro D1* to the *Rb1/Trp53/Rbl2* mouse. Interestingly, while loss *Ascl1* impedes appearance of tumors, inactivation of *NeuroD1* did not affect the development of SCLC [27]. The data further suggest that the preexisting SCLC models belong to the SCLC-A/Ascl1 high subtype. The mouse model representative of the NeuroD1 high is depicted in invasive tumors based on the activation of Myc and stabilization of Myc protein in addition to the loss of *Rb1* and *Trp53* (RPM mice) [73]. So far, no mouse models resulting in tumors representative of the SCLC-P or SCLC-Y groups have been generated. Presumably, future models will identify specific genetic events responsible for the development of these different tumor subtypes. However, a recent study using the RPM mouse model supports the conclusion that MYC drives (from a neuroendocrine cell of origin) the SCLC-A to SCLC-N to SCLC-Y subtypes in a temporal evolution. These data suggest that SCLC harbors cells in transcriptionally dynamic states of progression, and further depict the high intratumoral heterogeneity and plasticity of this disease [77]. This aspect of SCLC makes its subtyping even more complicated. 

Considering that different subtypes have different therapeutic vulnerabilities, these recent models related to subtyping in classification help to clarify the identification of biomarkers, development, and application of targeted therapies, and more personalized treatment options, another unmet need in SCLC. 

## 7. Imaging Techniques for the Detection and Diagnosis of Neuroendocrine Lung Tumors

Computed tomography (CT) and magnetic resonance imaging (MRI) are the most commonly morphological imaging modalities used in current diagnosis, location, and staging of lung neuroendocrine tumors [13,80,81,82], yet they only provide structural details. Therefore, more accurate tumor identification is being provided by functional imaging evaluation by means of Positron Emission Tomography (PET) scanning using different radiotracers. 

Neuroendocrine tumors express a high density of surface somatostatin receptors (SSTR), which allow imaging with radiolabeled somatostatin analogues (SSAs). The initial use of the tracer ^111^In-DTPA-Octreotide (OctreoScan®, OCT, MallinckrodtMedical, Petten, Holland) has recently been replaced by ^68^Ga-labeled somatostatin analogs: DOTA-Tyr3 octreotide (DOTA-TOC); DOTA-Tyr3/Thr8 octreotide (DOTA-TATE); and DOTA-1-Nal3 octreotide (DOTA-NOC). In addition to the advantages inherent in the physical characteristics of PET (spatial resolution, sensitivity, and quantitation), ^68^Ga-labeled ligands show a higher affinity for somatostatin receptor types 2 and 5 and more rapid blood clearance than the corresponding ^111^In-labeled tracers [83]. Besides tumor detection, SSTR imaging provides an estimation of receptor density and evidence of the functionality (internalization capacity) of receptors, which contribute important information for the assessment of whether the patient is a candidate for treatments which act on these receptors, such as SSA and PRRT. ^68^Ga-DOTA-peptide-PET/CT was found to be predictive of tumor response with PRRT using either ^90^Y- or ^177^Lu-labeled somatostatin analogs [81,84]. Several PET/CT studies comparing ^18^F-Fluorodeoxyglucose (FDG) and ^68^Ga-DOTA-peptides in patients with confirmed pulmonary neuroendocrine tumors conclude that well-differentiated TC and AC, which present high density of surface SSTRs, show greater avidity for ^68^Ga-DOTA-peptides, whereas the role of ^18^F-FDG PET in the evaluation of TC tumors is limited in diagnosis but not in prognosis. In any case, both tracers can have a complementary prognostic value in lung neuroendocrine tumors [85,86,87,88,89,90,91].

**Figure 2:**
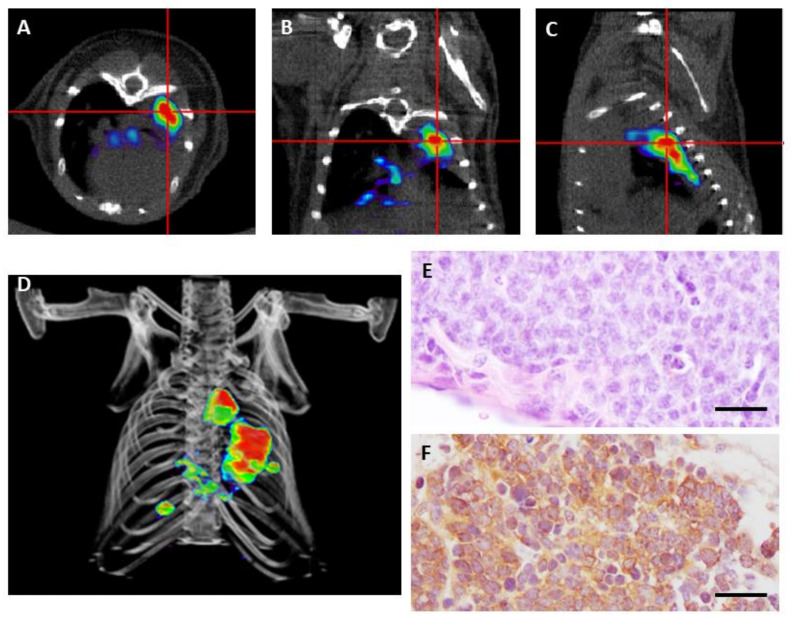
Positron emission tomography (PET) imaging with ^68^Ga-DOTA-TOC of a genetically engineered mouse bearing small cell carcinoma. (**A–C**) Transverse (**A**), coronal (**B**), and sagittal (**C**) views of combined micro PET/CT images where high tumor avidity for ^68^Ga-DOTA-TOC is detected. (**D**) 3-D image reconstruction showing growing tumors in the mouse lung. (**E**) Hematoxylin and eosin staining of a small cell carcinoma at necropsy. (**F**) Detection of somatostatin receptor 2 (SSTR2) by immunohistochemistry. Scale bars, 100 μm.

Lázaro et al. [22] performed ^18^F-FDG and ^68^Ga-DOTA-TOC PET in a GEMM model of human neuroendocrine lung cancer in an attempt to detect early lesions, monitor growth, and correlate imaging with histopathological findings. This study showed that ^68^Ga-DOTA-TOC was highly sensitive in detecting SCLC and LCNEC tumors, while there was an unexpected absence of ^18^F-FDG uptake despite their high proliferation index. The lack of ^18^F-FDG uptake in mice could be explained in part because of the low levels of the glucose transporter GLUT-1 in pulmonary neuroendocrine tumors [92] or the proximity of the heart to the selected region. Nevertheless, in human lung cancers, the SUV_max_ (Standarized Uptake Value) for ^18^F-FDG PET/CT for these highly aggressive tumors varies a lot among different patients, ranging from 2.8–25.4 [93]. Moreover, bronchial carcinoids have been traditionally considered as ^18^F-FDG PET/CT negative, but recent studies have found sensitivity of FDG-PET for the detection of carcinoids, both for the typical and atypical forms [87,94,95]. SUV_max_ for ^18^F-FDG PET/CT varied from 1.4–12.9 in twenty-five patients studied (24 TC and one AC) [96]. As a wide range of ^18^F-FDG avidity is observed, debate and controversy remain regarding its use for the diagnosis, classification, and choice of appropriate treatment regimens for lung neuroendocrine tumors [13]. In this scenario, ^68^Ga-DOTA-TOC could be an alternative for early detection and monitoring of primary lung neuroendocrine tumors, which is supported by the observations made in mouse models that do not consume ^18^F-FDG [22] (Figure 2).

Recent studies have reported that radiolabeled somatostatin receptor antagonists, although not internalized, show promise as superior agents to the currently used radiolabeled agonists for both imaging and PRRT. The binding characteristics of the antagonist showed a ten-times-higher number of binding sites compared to the agonist, possibly because the former interacts with predominately low-affinity receptor-sites [97,98]. In addition to ligands for SSTR, a number of other molecular imaging probes are in development, such as radiolabeled agonists interacting with the chemokine-receptor CXCR4, which is frequently overexpressed in proliferating SCLC tumors, and seems to be a promising new target for both diagnostic and therapeutic interventions [90,99]. To further prove functional evidence of these findings in a physiological in vivo experimental system mimicking the human disease, molecular imaging of GEMMs of lung neuroendocrine cancer emerges as an essential tool for the improvement of the identification and development of novel diagnostic or therapeutic drugs and to facilitate the translation of preclinical findings into the clinic. The ability to track these disease models long-term is therefore invaluable. Imaging of intact whole animals also facilitates the detection and investigation of systemic aspects of disease, such as cancer metastasis, which are not replicated in ex vivo systems [100].

## 8. Conclusions

Lung cancer neuroendocrine tumors share morphology and neuroendocrine properties. All of them are in urgent need of effective therapies, although they show marked differences in incidence and prognosis. This contrast is reflected in the diversity of mouse models available. While SCLC is one of the best represented diseases, with a variety of GEMMs successfully mimicking human neoplasia, there is a decided paucity of animal models of pulmonary carcinoids and LCNEC. As there has recently been a significant improvement in the dissection of molecular pathways and genomic studies for these understudied lung tumors, the evident lack of TC, AC, and LCNEC animal models could be hopefully addressed in the near future by using the increasingly accurate gene editing and targeting tools available to model lung cancer in mice, particularly for low-grade tumors whose incidences are increasing and metastasize to a high degree. Their shared expression of SSTR make these models especially suitable for the development and testing of molecular imaging techniques based on ^68^Ga-radiolabeled somatostatin analogs.

## Figures and Tables

**Table 1:** Lung neuroendocrine cancer models obtained by intratracheal adenoviral cre delivery.

Tumor Type		Genotype	Promoter	Major Phenotype	Ref.	Comments
**TC**		Rb1/Rbl1/Rbl2	CMV	TC	[21]	DHPN induction
**AC**		Rb1/Rbl1/Rbl2	CMV	AC	[21]	Urethane induction
**LCNEC**		Rb1/Rbl1/Trp53/Pten	CMV	LCNEC	[22]	First LCNEC mouse model described
**SCLC**		Rb1/Trp53	CMV	SCLC	[68]	First SCLC mouse model described
		Rb1/Trp53	CGRP, SPC	SCLC	[50]	Cells of origin of SCLC
	**Backbone *Rb1/Trp53 AND***					
		Rbl2	CMV	SCLC	[69]	Accelerated tumor development
		Rbl2	CGRP	SCLC	[54]	Different metastatic program
		Pten	CMV	SCLC/LCNEC/ ADC	[70]	Accelerated tumor development
		Pten	CGRP	SCLC	[76]	Accelerated tumor development
		Pten/Rbl1	K5	SCLC	[22]	Basal cell of origin of SCLC
		Mycl	CMV	SCLC	[71]	Accelerated tumor development
		Mycl	CMV	SCLC	[72]	Heterogeneity and differential sensitivity to chemotherapy
		Myc ^T58A^	CGRP	SCLC	[73]	SCLC-N subtype
		Myc ^T58A^	CGRP	SCLC	[77]	Multiple subtypes present in a tumor
		Nfib	CMV	SCLC	[75]	Short latency and enhanced metastases
		Rbl2/Notch2	CMV	SCLC	[17]	Role of Notch as tumor suppressor in SCLC
		Rbl2/NeuroD1	CMV	SCLC	[27]	SCLC-A subtype
		FgFr1	K14	SCLC/ rare ADC	[53]	Context dependent effect of Fgfr1

TC: typical carcinoma; AT: atypical carcinoma; LCNEC: large cell neuroendocrine carcinoma; SCLC: small cell carcinoma; ADC: adenocarcinoma.
